# 

**Published:** 1889-10-26

**Authors:** 


### THE HOSPITAL

A WEEKLY INSTITUTIONAL JOURNAL OF
**Science, Medicine, Nursing & Philanthropy.**

### SATURDAY, OCTOBER 26th, 1889.

**Conscience** - 49

**Words of Consolation**—Joy - 50

**The Work of the Hospitals Association.** By Dr. Bristowe, F.R.S., President - 51

**The Doctor's Pulpit.**—Physiological Candles - 53

**The Hospital Workshop.** XII.—The General Hospital at Birmingham - 54

**Everybody's Page** - 55

**Annotations.**—Nursing at Netley—The Matron's Powers—Hair Dye - 58

**Notes and News** - 56

**The Practitioner's Outlook and Retrospect:**

Medicine—Phthisis—Ovarian Disease—Heredity—Cancer - 59

Surgery—Lithotomy—Removal of Accessory Limb—Necrosis of the Jaw - 60

Therapeutics—Sea-Sickness, 60; Salol - 61

Books Received - 61

**The Royal Chest Hospital Scandal.**—A Statement of the Facts - 61

**Scraps and Gleanings** - 64

### EXTRA SUPPLEMENT—THE NURSING MIRROR.

**En Passant.**—The Pension Fund—Christmas Decorations—Short Items—Nurses and Temperance—Troublesome Probationers—The Hope Infirmary - xvii
**Cottage Hospital Matrons.** II.—Their Nursing Duties xviii
**In Loving Memory** - xviii
**An Exhibition of Nursing Uniforms.**—Final Particulars - xviii
**Everybody's Opinion.**—Superannuated at Forty-Three xviii
**Notices** - xviii
**The Two Surgeons** - xix
**Death in Our Ranks.**—Our Nurse - xx
**Appointments—Notes and Queries** - xx
**Vacancies** - xx
**Amusements and Relaxation** - xx

